# SPINDILOMETER: a model describing sleep spindles on EEG signals for polysomnography

**DOI:** 10.1007/s13246-024-01428-7

**Published:** 2024-05-31

**Authors:** Murat Kayabekir, Mete Yağanoğlu

**Affiliations:** 1 Department of Physiology, Medical School, Atatürk University, 25240 Erzurum, Turkey; 2 Department of Computer Engineering, Faculty of Engineering, Atatürk University, Erzurum, Turkey

**Keywords:** Sleep electrophysiology, Sleep spindle, Machine learning, Easy diagnosis

## Abstract

This paper aims to present a model called SPINDILOMETER, which we propose to be integrated into polysomnography (PSG) devices for researchers focused on electrophysiological signals in PSG, physicians, and technicians practicing sleep in clinics, by examining the methods of the sleep electroencephalogram (EEG) signal analysis in recent years. For this purpose, an assist diagnostic model for PSG has been developed that measures the number and density of sleep spindles by analyzing EEG signals in PSG. EEG signals of 72 volunteers, 51 males and 21 females (age; 51.7 ± 3.42 years and body mass index; 37.6 ± 4.21) diagnosed with sleep-disordered breathing by PSG were analyzed by machine learning methods. The number and density of sleep spindles were compared between the classical method (EEG monitoring with the naked eye in PSG) (‘method with naked eye’) and the model (SPINDILOMETER). A strong positive correlation was found between ‘method with naked eye’ and SPINDILOMETER results (correlation coefficient: 0.987), and this correlation was statistically significant (p = 0.000). Confusion matrix (accuracy (94.61%), sensitivity (94.61%), specificity (96.60%)), and ROC analysis (AUC: 0.95) were performed to prove the adequacy of SPINDILOMETER (p = 0.000). In conclusion SPINDILOMETER can be included in PSG analysis performed in sleep laboratories. At the same time, this model provides diagnostic convenience to the physician in understanding the neurological events associated with sleep spindles and sheds light on research for thalamocortical regions in the fields of neurophysiology and electrophysiology.

## Introductıon

The synaptic electrical activity and voltage changes [non rapid eye movement (NREM) sleep, characterized by high amplitude, low-frequency brain waves, and rapid eye movement (REM) sleep, defined by low amplitude, higher frequency EEG activity, with mixtures of these occurring during transitional phases] that occur in the brain during sleep each night enable the reintegration of sensory and motor networks throughout the body [1–6]. Sleep is a physiological process organized in stages (NREM1, NREM2, REM, NREM3). The K-complex and sleep spindle are the cornerstones of the sleep-EEG microstructural architecture. These electrophysiological microelements play an important role in understanding sleep’s neurophysiological and functional aspects [2, 7]. Sleep spindles are EEG rhythms especially prominent during NREM 2 [6]. They are oscillatory EEG activities in the sigma frequency band (∼11–16 Hz) of fusiform morphology that last around 0.5–3 s [8–10]. Two types of sleep spindles have been recognized; fast spindles at 14–15 Hz maximal in centroparietal regions and slow spindles at 12–13 Hz predominant in frontal areas [7, 11, 12]. Sleep spindles are thalamocortical oscillations [13, 14] with the physiological potential to facilitate neuroplasticity [15–17]. Sleep spindle characteristics such as spindle density, frequency or amplitude are trait-like individual characteristics with genetic and anatomical underpinnings [18–21]. Sleep spindles are electrophysiological characteristics with a function very closely related to cognition [22] and intelligence [23], with normal aging processes [24, 25]. Changes in the number and intensity of sleep spindles observed in EEG may indicate a malfunction in the thalamocortical circuit (e.g., schizophrenia, epileptic seizures, Parkinson’s, Alzheimer, mental retardation, abnormal maturation but also with recovery processes as in post brain stroke) [8, 26–29]. In recent years, the pace of sleep EEG studies that associate electrophysiological activity to cognition and disease has increased [30]. Computers detect sleep spindles automatically, but the results must be evaluated by visual-scoring experts and sleep physicians. Accurate recognition of microelements in EEG, understanding their electrophysiological properties, and interpreting their number and intensity provide important information for brain health. Although a limited number of automatic sleep spindle counters have been designed in the literature [31–35], they have not yet taken their place as a basic analysis tool in interpreting PSG reports. In sleep analysis laboratories, PSG is the gold standard diagnostic method for recognizing sleep physiology and disorders. This article aims to present a model called SPINDILOMETER, which we propose to be integrated into PSG devices for researchers focusing on electrophysiological signals in PSG as well as, physicians, and technicians practicing sleep in clinics, by examining the methods of the sleep EEG signal analysis in recent years.

## Materials and methods

This study was carried out in the Sleep Disorders Center, Electrophysiology Laboratory in Erzurum Regional Training and Research Hospital. Local ethics committee of the Atatürk University Medical School approved this study with an Approval Number of 06-29/28.05.2020. National Utility Model and Patent Project Number: 2023/12365. Date: 23.03.2023. The laboratory rooms were designed in accordance with the American Academy of Sleep Medicine (AASM) guidelines.

### Study participants

The study was planned according to Helsinki Declaration. EEG signals of 72 volunteers, 51 males and 21 females (age; 51.7 ± 3.42 years and body mass index; 37.6 ± 4.21) diagnosed with sleep-disordered breathing by PSG were analyzed. The mean total sleep time of the volunteers during one night’s sleep was 6 ± 2.37 h.

### Experimental recordings

All patients underwent a full‐night laboratory PSG using the Grass Technologies PSG system (TWin 4.5.3, USA). During sleep, polysomnography consists of recording different physiological and pathophysiological parameters for period of 6 h or longer throughout the night: these are reported after evaluations by a clinical physiologist whose field of study is sleep medicine and neurophysiology (M.D.). Electrophysiological signals recorded during sleep and wakefulness throughout the night are “Electroencephalogram (EEG), electromyogram (EMG; jaw, arm and leg), electrooculogram (EOG), electrocardiogram (ECG), snoring, oro-nasal airflow (lt/s), chest and abdominal movements (respiratory effort recordings), oxygen saturation, body position and real time-video-image recordings”. F4-M1, C4-M1, and O2-M1 and F3-M2, C3-M2, and O1-M2 sleep EEG channels allow for recordings from six different locations on the head with standard PSG [1, 3, 9, 36].

### Experimental protocol

**Fig. 1:**
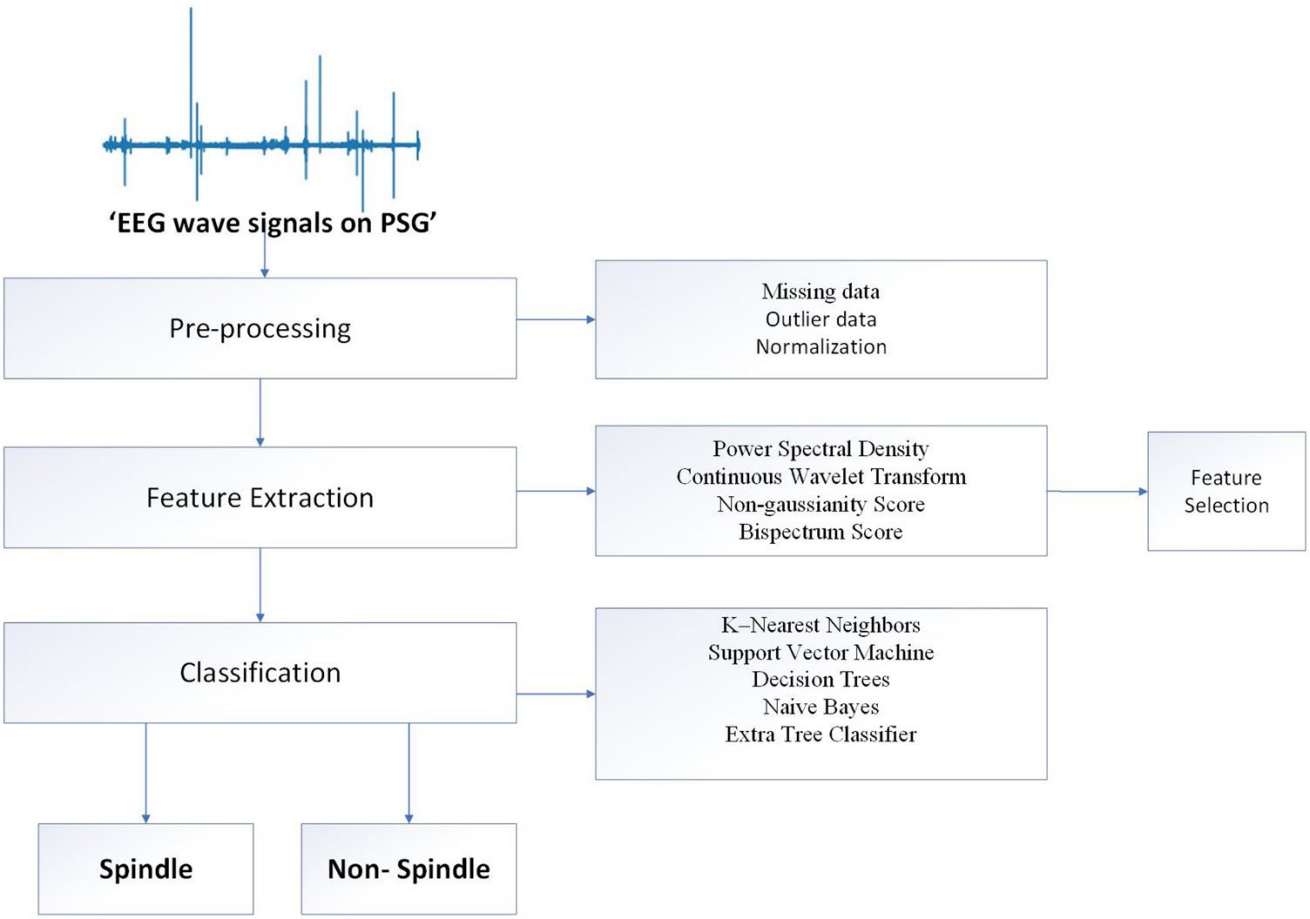
Experimental protocol

While sleep breathing disorders diagnoses of the volunteers were established according to AASM together with the physiology specialist (M.D.) responsible from the laboratory, the electrophysiological properties, number and density of sleep spindles in the EEG were examined and counted at least 3 times for each of the 6 channels with the naked eye (EEG monitoring with naked eye in PSG), (‘method with naked eye’). EEG signal waves (sleep spindles) with fusiform morphology in each of six different EEG channels (F4-M1, C4-M1 and O2-M1, F3-M2, C3-M2, and O1-M2) lasting approximately 0.5–3 s at 11–16 Hz were analyzed by machine learning methods (SPINDILOMETER) (Fig. 1). The number and density of sleep spindles were compared between the classical method (‘method with naked eye’) and the model (SPINDILOMETER).

### Experimental setting

The idea of using machine learning methods to analyze sleep spindles and reveal clues about basic and clinical events related to thalamocortical activity in the brain is the main inspiration for the development of SPINDILOMETER. SPINDILOMETER contains units that analyze the frequency and amplitude values of EEG signals in PSG. SPINDILOMETER stores these values, uses them, and decides whether a sleep spindle exists [sigma frequency band (~ 11–16 Hz)] (Fig. 1). Care was given to use the latest machine learning methods in developing this model. Table 1 describes the algorithm for the model: first, the missing data were normalized by replacing it with the mean value. Next, four different feature extraction algorithms were applied to this data: “Power Spectral Density, Continuous Wavelet Transform, Non-gaussianity Score and Bispectrum Score Features (X_d_).” Then, appropriate features were determined by the feature selection process. In the final stage, the number and characteristics of sleep spindles were revealed by using classification algorithms.

**Table 1:**
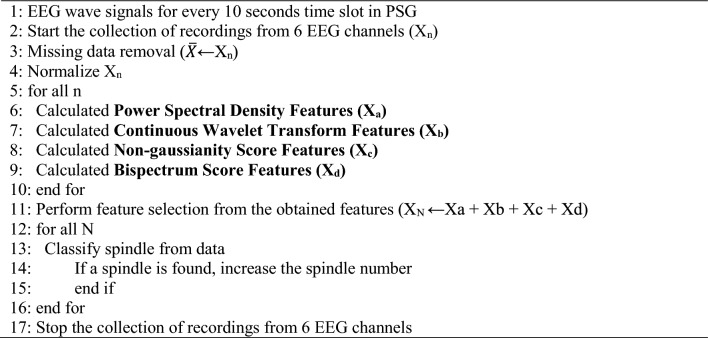
Algorithm for SPINDILOMETER

### Data editing and analysis

#### A general technical approach to sleep spindle extraction methods in EEG signals

**Fig. 2:**
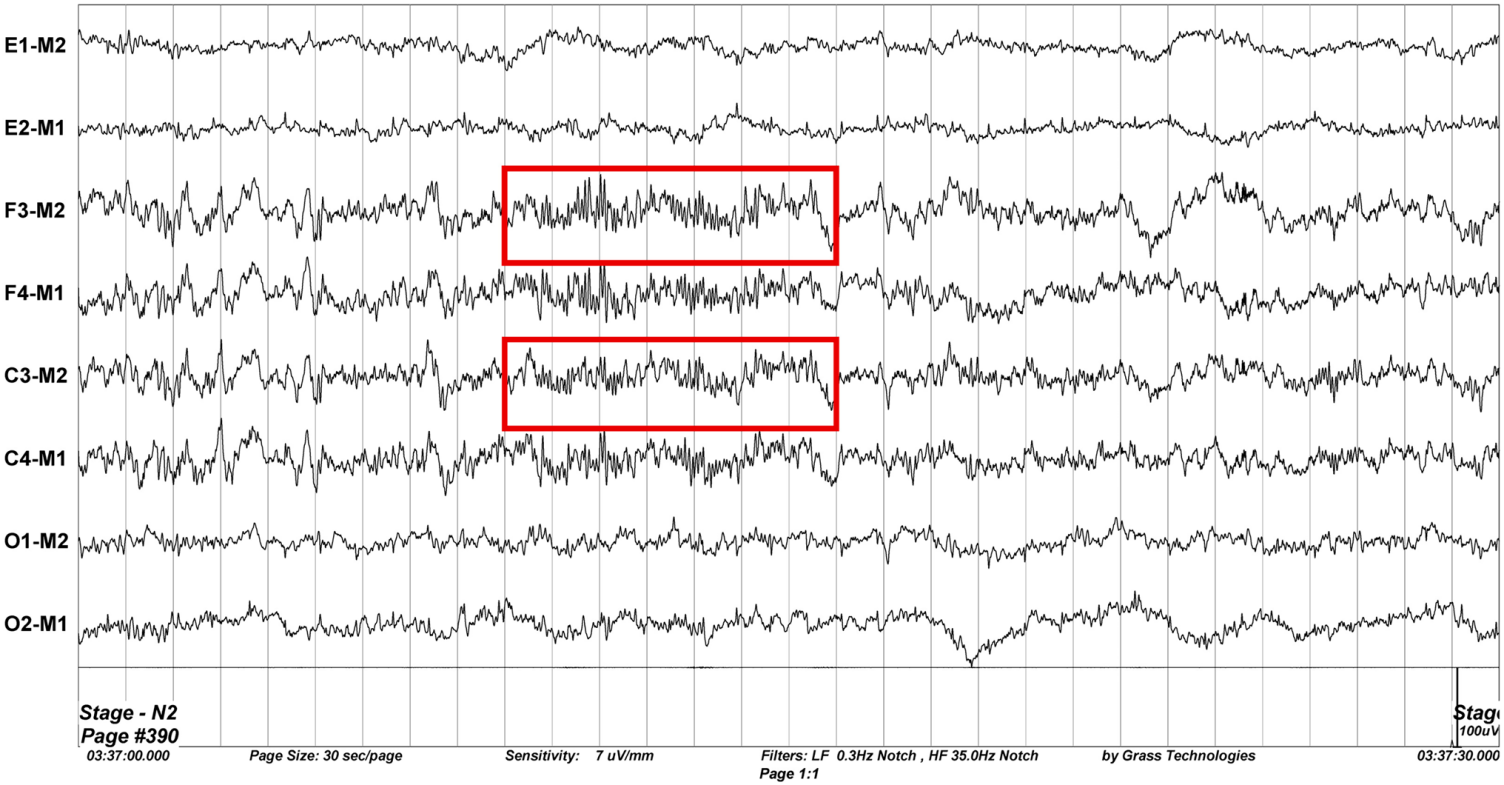
EEG recording in polysomnogram “Grass Technologies PSG system, TWin 4.5.3, USA”. Sleep spindles are seen in red boxes at stage N2. EEG, electroencephalography; 6 EEG channels: Frontal, F3 M2, F4 M1; Centro-parietal, C3 M2, C4 M1; Occipital, O3 M2, O4 M1; PSG, polysomnography; N2, NREM2, Non-Rapid Eye Movement stage 2

On each of the channel signals were analyzed by using computer-based electrophysiological signal analysis methods. The process consisted of “Pre-processing, Feature extraction, Feature selection, and Classification”. We applied highly reliable signal analysis methods used in computer science to sleep medicine. For this reason, we used a wide spectrum of multiple (9 specifics) signal analysis methods for the analysis of EEG signal waves in PSG. The EEG wave signals of a sample PSG recording from the study (Fig. 2) and the relationship between the methods applied to these signals and the EEG signals are explained below.

### Pre-processing of EEG signals for SPINDILOMETER

Six-channel EEG wave signals were obtained from the electrophysiological signal recordings obtained from PSG, and were accepted as a data set. EEG wave signals were analyzed for each PSG epoch (30 s periods) during at least 6 h of sleep for each volunteer. We tried to see the details by dividing each age into 10-s segments to better understand the EEG waves. Seventy percent of the data set was split into two as training and 30% as test phase. Sleep spindles detected by the researcher (M.D.) who reported the PSG were estimated in the testing phase. The technical procedure in the data preprocessing phase was as follows: (1) Missing data were identified and filled with mean values. (2) Outlier data were identified and subjected to normalization. Minimum–Maximum Normalization: one of the most common methods used to reduce the differences between the data and to normalize the data. (a) the minimum value of the amplitude and frequency values of the EEG waves was set as ‘0’ and the maximum value as ‘1’, (b) all values between the minimum and maximum values were converted to ‘decimal numbers.’

### Feature methods used for SPINDILOMETER and their correlation with EEG wave signals

#### Power spectral density (PSD)

Frequency is a characteristic of EEG signals. PSD measures the power content of a signal versus frequency or the energy density of a signal at different frequencies. In the time domain, it is difficult to find distinctive features of EEG signals, but in the frequency domain, PSD finds similarities and differences as the maximum values are known [37]. Since PSD is the energy of the signal per frequency, it is defined as the Fourier transform of an EEG signal’s autocorrelation function (A(ξ)). Its formula is as follows (ξ is the spatial shift, Ω is the number of waves) [38]:

$$\Phi \left(\Omega \right)=\frac{1}{2\pi }\underset{-\infty }{\overset{+\infty }{\int }}A(\xi ){e}^{-i\Omega \xi }d\xi $$

#### Continuous wavelet transform (CWT)

EEG waves are chaotic bio-signals. CWT allows provisioning an over-complete signal representation by allowing the translation of wavelets and scale parameters to change continuously. Thus, it generates a large number of wavelet coefficients. These coefficients can be used as features. The scaling of a wavelet is expressed as its compression or expansion. Unlike the Fourier transforms, the time–frequency window of the CWT is adjustable. Its formula is as follows (b is the shift factor, α is the scale factor, t is the time, and f(t) is the signal vector of interest) [39]:

$$CWT=\frac{1}{\sqrt{\alpha }}{\int }_{-\infty }^{+\infty }f\left(t\right)\varphi \left(\frac{t-b}{\alpha }\right)dt$$

#### Non-Gaussianity score (NGS)

This score was used to understand the distribution of EEG signal characteristics (amplitude, frequency). NGS indicates the non-Gaussianity of a given data segment. This method made it easy to measure the deviation of the EEG signals in each epoch of the PSG from the Gaussian model. The formula is as follows (p and q are the normal probability plots of the reference and analyzed data, respectively) [40]:

$$NGS=1-\left(\frac{{\sum }_{j=1}^{N}{\left({q}_{j}-p\right)}^{2}}{{\sum }_{j=1}^{N}{\left({q}_{j}-\overline{\overline{q}}\right)}^{2}}\right)$$

#### Bispectrum score (BGS)

The BGS, the 3rd order spectrum of the signal, is known as the bispectrum. Unlike the autocorrelation-based power spectrum (2nd order statistics), the bispectrum preserves Fourier phase information. It allows in-depth analysis of the EEG wave signals at each epoch in PSG. Its formula is as follows (ω1 = ω2 = ω; through the defined diagonal slice P(ω), the information available in the bispectrum is captured) [41]:

$$BGS=\frac{{\int }_{\omega 1}^{\omega 2}P(\omega )}{{\int }_{w3}^{w4}P(\omega )}$$

### Feature selection for SPINDILOMETER

This process aims to simplify the number of attributes. Attribute selection focuses on finding an optimal subset of attributes (defining which attribute is more important). In our study, filtering methods were used in the attribute selection phase. The filtering method examines each attribute’s susceptibility in the dataset to each classification [42–44].

### Classifiers used for SPINDILOMETER and their relation with EEG wave signals

#### Classification

Classification is the process of determining to which class an unknown pattern belongs with the help of a classifier that uses the features of that pattern as input. This study classified EEG signals using the most appropriate machine learning methods to identify sleep spindles among the EEG signals during PSG [45, 46].

#### K-nearest neighborhood (KNN)

KNN is a learning algorithm and its goal is to perform a classification on the existing training data when a new sample is received [47]. KNN was preferred in this study due to the simplicity of the method and its high accuracy when used on EEG data. KNN method is a simple and effective method.

#### Support vector machine (SVM)

Support vector machines are a highly effective, simple machine-learning method for classification problems in data sets where the patterns between variables are unknown. It minimizes the classification error by selecting the line with the highest margin (necessary for discriminating sleep spindle wave signals). Therefore, SVM has facilitated the rapid and reliable extraction of the characteristic wave signals of the sleep spindle for the SPINDILOMETER model [48, 49].

#### Decision trees (DT)

Decision trees are one of the supervised machine learning algorithms [50]. The purpose of using the decision tree algorithm in this study is to learn decision rules extracted from the features of EEG wave signals and then develop a model that can predict the value of the target variable (sleep spindles).

#### Naive Bayes

The *Naive Bayes* algorithm helped us to classify the features of EEG wave signals as it is easy to apply and understand in large data sets: this method works by assuming that the presence of an attribute in a given class is not related to the presence of any other attribute [51]. This method provided us with important data on the probability of discovering specific sleep spindle wave signals that we tried to find among the EEG wave signals from 6 channels, which is the dataset of our study.

#### Extra tree classifier (ETC)

The ETC differs from other tree-based ensemble methods for two main reasons: (1) it separates nodes by choosing breakpoints completely randomly, and (2) it uses all learning samples to grow the trees [52]. With the ETC ensemble implementation, the entire sample of EEG wave signals was used and classified.

### Confusion matrix tables comparing sleep spindles numbers calculated by both methods

In the model developed in this study, the EEG waves of PSG recordings collected from all volunteers were divided into 10-s time slices to prove that each sleep spindle accurately identifies the electrophysiological wave signal. The confusion matrix was used to measure the success of the model. In order to see the effectiveness of a classifier, the prediction accuracy of the classifier should be measured after the training phase is completed. In this study, after the confusion matrix was created, the performance of the prediction models were compared using the criteria of accuracy, sensitivity, precision and F1 score. The confusion matrix is a table used to show how much of the predicted value of the classification model created as a result of machine learning algorithms matches the actual class value. It summarizes and visualizes the performance of the classification algorithm. We tried to understand the success of the SPINDILOMETER algorithm in identifying sleep spindles by means of performance measures for results obtained from each volunteer. Performance measures applied to SPINDILOMETER are the following: (a) Accuracy is calculated as the number of all correct predictions divided by the total number of the dataset. (b) Sensitivity and Recall are calculated as the number of true positive predictions divided by the total number of true positive classes. (c) Precision is the number of true positive predictions divided by the total number of positive predictions. (d) F1-score is the harmonic mean of Precision and Recall.

### Comparison of ‘method with naked eye’ and SPINDILOMETER methods with ROC analysis

One of the statistical techniques commonly used to evaluate the performance of a classifier is the receiver operating characteristics (ROC) curve. ROC curves provide a visual approach to judge the efficiency of a classifier. In this study, in addition to the performance measures obtained from the confusion matrix, the area under the ROC curve (ROC–AUC) value was also calculated and evaluated. The ROC-AUC score was chosen as an evaluation criterion to determine the degree of separability. It measures the classification performance of the model at all possible thresholds. With this metric, we evaluate how well the model works when discriminating between sleep spindle and non-sleep spindle electrophysiological wave signals. If the ROC-AUC value is high, we can say that the model is successful.

### Statistical analysis

The number and density of sleep spindles were determined from the EEG recordings of the physician who examined the real-time PSG during sleep with the naked eye (classical method, ‘method with naked eye’) and SPINDILOMETER and compared using the ‘SPSS 22 for Windows program. The Intra-class Correlation Coefficient test was used to analyze the results. In this comparison, p < 0.001 was considered statistically significant. The accuracy of our algorithm was analyzed with confusion matrix tables. Finally, two methods (‘method with naked eye’ and SPINDILOMETER) were compared by ROC analysis to prove that our algorithm can successfully identify each sleep spindle count in the shortest time interval (10 s).

## Results

### Results of the classifiers used for the development of the SPINDILOMETER

The highest values belong to the KNN algorithm. The KNN algorithm achieved 94.61% accuracy, 92.47% sensitivity, and 96.60% specificity (Table 2).

**Table 2:** Results of the classifiers (algorithms) used for the development of the SPINDILOMETER

Algorithm	Accuracy (%)	Sensitivity (%)	Specificity (%)	F1 score (%)
KNN	94.61	92.47	96.60	94.30
SVM	94.09	91.80	96.23	93.76
DT	92.31	89.07	95.47	91.97
NB	91.60	89.46	93.59	91.11
ETC	93.53	91.12	95.80	93.18

*KNN* K-nearest neighborhood, *SVM* support vector machine, *DT* decision trees, *NB* Naive Bayes, *ETC* extra tree classifier

### Comparison of ‘method with naked eye’ and our model in terms of sleep spindle detection

**Fig. 3:**
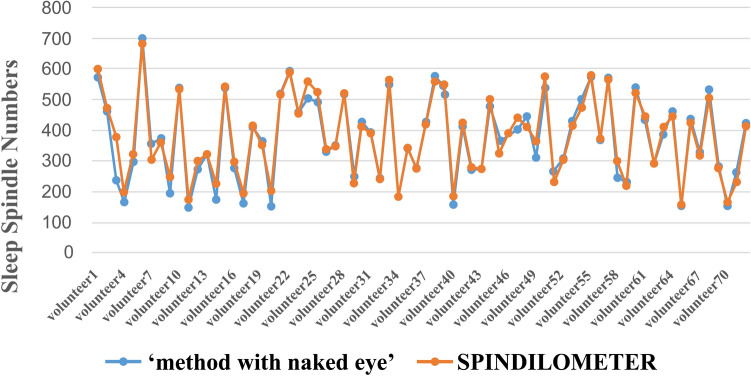
The amount of sleep spindles detected by both models for each volunteer appears graphically. The blue curve ‘method with naked eye’, the orange curve graphically shows the SPINDILOMETER’s sleep spindle numbers, and the table below this figure shows the sleep spindle numbers numerically

**Fig. 4:**
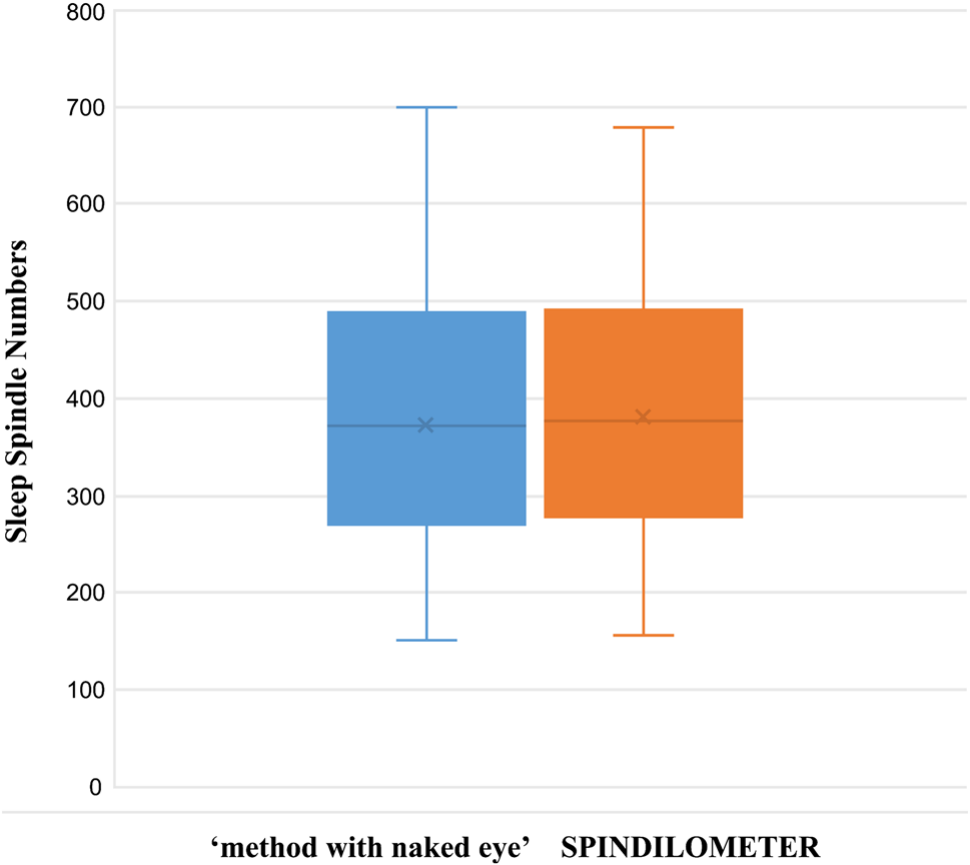
The comparison of the number of sleep spindles calculated by ‘method with naked eye’ and SPINDILOMETER methods as a histogram

The number of sleep spindles for each volunteer was determined separately for both models. A strong positive correlation (correlation coefficient: 0.987) was found between ‘method with naked eye’ and SPINDILOMETER results, and this correlation was statistically significant (Intra-class Correlation Coefficient Test: p = 0.000) (Tables 3, 4, Figs. 3, 4).

**Table 3:** There was a strong positive correlation between ‘method with naked eye’ (correlation coefficient: 0.987) and SPINDILOMETER results, and this relationship was statistically significant (intraclass correlation coefficient test: p < 0.001)

	Intraclass correlation^b^	95% confidence interval		F test with true value 0			
		Lower bound	Upper bound	Value	df1	df2	Sig.
Single measures	0.975^a^	0.960	0.985	83,108	71	71	0.000
Average measures	0.987^c^	0.979	0.992	83,108	71	71	0.000

**Table 4:** Numerical values found by the ‘method with naked eye’ and SPINDILOMETER models during one night sleep

n = 72	‘Method with naked eye’—sleep spindle numbers	SPINDILOMETER—sleep spindle numbers
Min	151	157
Max	700	680
Med	369	367
Standard dev	131.84	127.88

### Findings of confusion matrix tables comparing sleep spindle numbers calculated by both methods

**Fig. 5:**
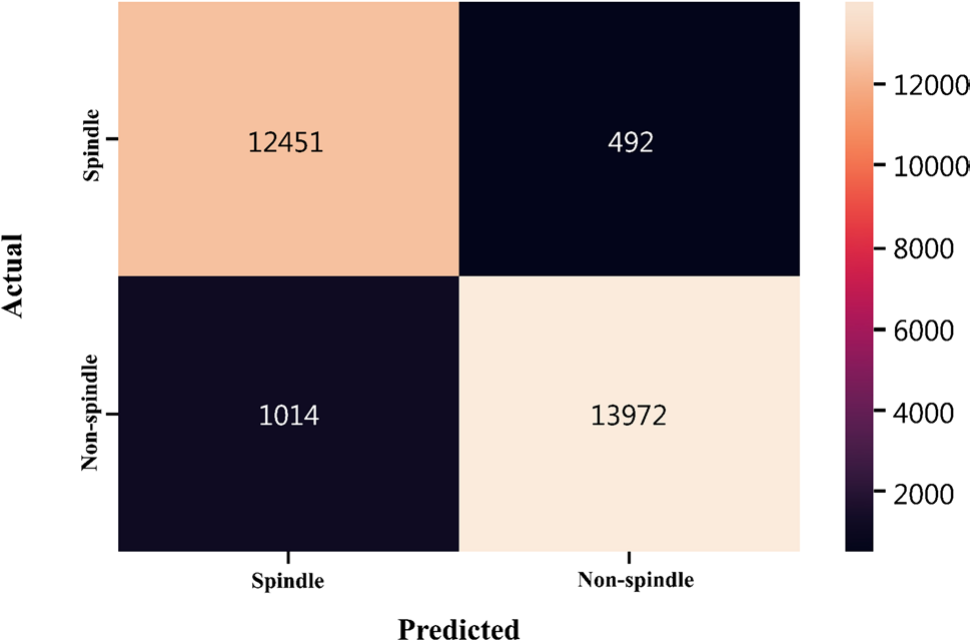
Actual (calculated by ‘method with naked eye’) and predicted (calculated by SPINDILOMETER) numerical values of sleep spindles for all volunteers. As concerns the values calculated for volunteers, when 10 s time frames are taken into consideration, sleep spindles are not defined 13,972 times by both methods (TN); defined by SPINDILOMETER 1014 but not with ‘method with naked eye’ for 1014 times (FP); were defined by either of the methods for 12,541 times (TP); defined 492 times by ‘method with naked eye’ despite not being defined by SPINDILOMETER (FN) [According to this matrix, Sensitivity: 0.9247 Specificity: 0.9660 Precision: 0.9660, Accuracy: 0.9461, F1-Score: 0.9430 and Matthews Correlation Coefficient: 0.8925 were obtained for the model SPINDILOMETER]. *EEG* electroencephalography, *PSG* polysomnography, *TN* true negative, *FP* false positive (FP), *TP* true positive, *FN* false negative

Numerical values pertaining to the methods were accepted as actual for ‘method with naked eye’ and predicted for SPINDILOMETER. If sleep spindles are identified in a 10 s time frame it is marked as 1; not as 0 (Fig. 5: demonstrates the confusion matrix for all of the volunteers).

Table 5 demonstrates that the performance score for each volunteer is statistically significantly high.

**Table 5:** Performance measures for all the volunteers in the data set were obtained by taking 10 s time frames into consideration

Volunteer	Accuracy	Precision	Recall	F1 score
Volunteer 1	94.61	92.47	96.2	94.3
Volunteer 2	93.81	92.05	95.42	93.71
Volunteer 3	88.47	86.25	89.47	87.83
Volunteer 4	88.73	95.3	82.84	88.64
Volunteer 5	90.07	93.69	87.22	90.34
Volunteer 6	87.32	90.31	82.73	86.35
Volunteer 7	87.98	84.89	91.1	87.88
Volunteer 8	86.59	89.33	84.6	86.9
Volunteer 9	87.93	92.15	83.94	87.85
Volunteer 10	94.59	96.3	92.71	94.47
Volunteer 11	96.46	98.13	94.75	96.41
Volunteer 12	95.64	95.13	96.1	95.61
Volunteer 13	86.09	88.96	83.98	86.4
Volunteer 14	96.94	98.03	96.29	97.15
Volunteer 15	97.94	98.01	97.83	97.92
Volunteer 16	94.61	95.02	93.89	94.45
Volunteer 17	93.57	94.16	92.36	93.25
Volunteer 18	97	99.66	94.83	97.19
Volunteer 19	93.82	92.92	94.29	93.6
Volunteer 20	94.67	94.94	94.06	94.5
Volunteer 21	95.64	93.66	97.67	95.62
Volunteer 22	97.21	96.62	97.66	97.14
Volunteer 23	93.46	93.14	94.25	93.69
Volunteer 24	96.66	94.12	99.27	96.63
Volunteer 25	94.6	93.54	95.3	94.41
Volunteer 26	93.12	94.4	92.29	93.33
Volunteer 27	95.73	96.44	94.5	95.46
Volunteer 28	96.21	98.03	94.61	96.29
Volunteer 29	98.74	99.63	97.85	98.73
Volunteer 30	95.82	97.19	94.35	95.75
Volunteer 31	94.02	94.82	93.61	94.21
Volunteer 32	97.36	95.56	99.15	97.32
Volunteer 33	98.04	98.35	97.81	98.08
Volunteer 34	96.85	97.86	95.64	96.73
Volunteer 35	93.82	94.59	93.33	93.96
Volunteer 36	96.72	97.65	96.04	96.84
Volunteer 37	96.48	98.39	94.82	96.57
Volunteer 38	96.16	97.7	94.36	96
Volunteer 39	96.2	97.93	94.51	96.19
Volunteer 40	96.15	97.91	94.3	96.07
Volunteer 41	95.29	96.64	94.14	95.37
Volunteer 42	95.54	96.96	94.25	95.59
Volunteer 43	94.79	95.89	94.05	94.96
Volunteer 44	97.89	97.05	98.76	97.9
Volunteer 45	95.23	96.56	94.55	95.55
Volunteer 46	92.68	90.21	95.38	92.72
Volunteer 47	92.98	94.69	91.45	93.04
Volunteer 48	94.06	92.14	95.91	93.99
Volunteer 49	98.06	98.36	97.83	98.09
Volunteer 50	94.22	93.47	95.44	94.44
Volunteer 51	95	97.86	92.28	94.99
Volunteer 52	94.8	94.24	95.3	94.77
Volunteer 53	92.49	91.5	94.01	92.74
Volunteer 54	98.2	96.99	99.42	98.19
Volunteer 55	93.29	95.86	90.94	93.33
Volunteer 56	97.62	97.33	97.85	97.59
Volunteer 57	94.6	94.68	94.2	94.44
Volunteer 58	93.72	91.98	95.38	93.65
Volunteer 58	98.02	97.68	98.38	98.03
Volunteer 60	93.37	94.03	92.14	93.07
Volunteer 61	95.59	98.96	92.52	95.63
Volunteer 62	94.1	95.71	92.57	94.12
Volunteer 63	94.52	94.59	94.42	94.51
Volunteer 64	94.88	93.43	95.81	94.6
Volunteer 65	95.11	96.15	94.41	95.27
Volunteer 66	95.35	92.79	98.05	95.34
Volunteer 67	97.09	96.57	97.81	97.18
Volunteer 68	95.01	91.27	98.87	94.92
Volunteer 69	97.62	96.35	99.11	97.71
Volunteer 70	95.14	95.73	94.33	95.03
Volunteer 71	97.64	98.35	97.1	97.72
Volunteer 72	91.49	92.39	90.91	91.64

### Findings of both methods compared with ROC analysis

**Fig. 6:**
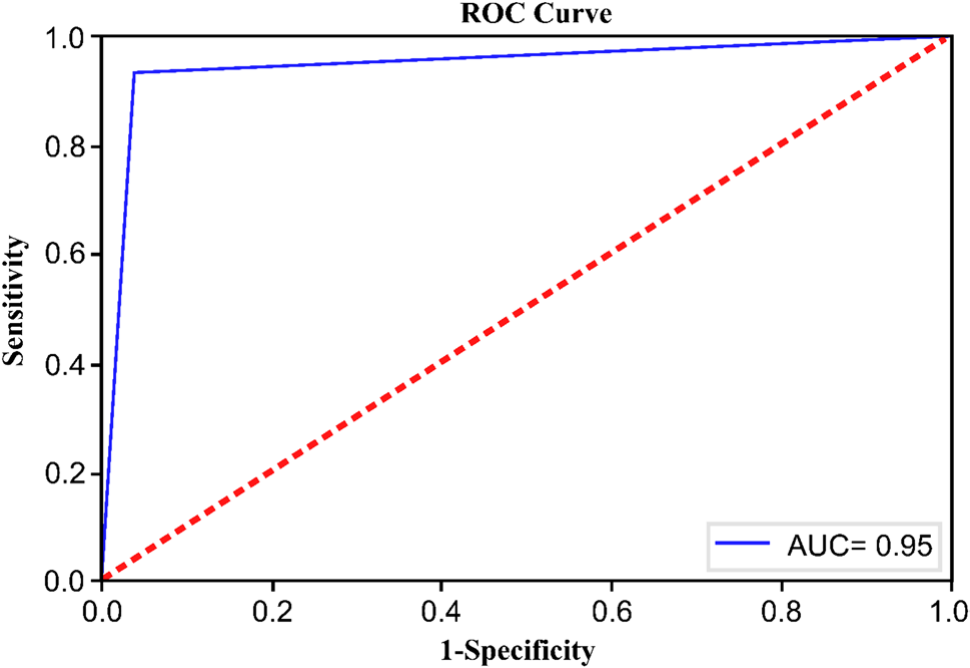
ROC curve—demonstrates the relationship between the sensitivity and specificity of ‘method with naked eye’ and SPINDILOMETER test results. Red dashed line represents a test that does not have correct discriminative features; the area under the curve is 0.5. For the best test performance, this needs to be 1. As we go upwards from the red dashed line, the increasing slopes of the appearing curves show us that the discriminative capacity of the diagnostic test under consideration has increased. Therefore, in order to talk about a high sensitivity and high specificity for a diagnostic test we anticipate that the area under the ROC curve should be 0.5–1.0

In our study, ROC curves were drawn to make a statistical comparison of the performances of the two methods and to delineate the relationship between the sensitivity and specificity of the two methods in detail. Furthermore, by showing the size of AUC, high discriminative performance of sleep spindle definitions pertaining to our method was demonstrated (Fig. 6).

The blue curve is a ROC curve with which we compare SPINDILOMETER and ‘method with naked eye’ for sensitivity and specificity; having a high slope and having an area under the curve (AUC) of 0.5–1 proves us that our method that is the subject matter of our study has a high sensitivity, high specificity and high precision (Fig. 6).

## Discussions

This study calculates the number and density of sleep spindles in PSG, an important diagnostic tool in medicine used in the fields of sleep neurophysiology and neuroscience, using optimal machine-learning methods with high success rates, and proposes a model called SPINDILOMETER to be integrated into PSG (PSG^+^). During the study, electrophysiological signals of the sleep spindle, one of the cornerstones of the sleep-EEG microstructural architecture, were analyzed to understand the connections between sleep spindles and the neurophysiological and functional aspects of sleep. In addition, this study provides a perspective on the recent scientific studies on sleep spindle and EEG signal analysis.

### Sleep spindles analysis for physiological studies and brain pathophysiology

Sleep spindle waveforms are named “spindles” and are inspired by wool spinning tools. They are 10–15 Hz sinusoidal cycles that appear as burst-like sequences in NREM 2 in EEG [53]. Their generation takes place between the thalamoreticular nucleus (TRN) and thalamocortical (ThCx) generator-neuronal circuits, which are closely interconnected. As deep sleep progresses, inhibitory neurons in the TRN begin to fire in bursts, causing massive generation of inhibitory postsynaptic electrical potentials in ThCx neurons. The precise physiological mechanisms in these regions (TRN-ThCx), referred to as neuronal generator circuits, are considered to be a critical line between deep sleep and wakefulness modes of the brain (under the control of the reticular activating system in the brainstem) [8, 25–28]. The model SPINDILOMETER, which we intend to make more and more functional, is a physiological detector of this critical region. In recent studies, the sleep spindles that appear in the EEG, which seem to originate from these sensitive generator circuits (TRN-ThCx), has been found to cause the following physiological mechanisms: (1) They facilitate neuroplasticity [13, 14]. (2) Since TRN-ThCx oscillations significantly change the activity in the gray matter of the cortex (a large number of neuronal circuits in the white matter that process incoming information and release new information), they play a decisive role in the control of movement, memory, and emotion [15–17]. (3) Sleep spindles show a characteristic profile throughout life (from the early postnatal period to adolescence and aging) in parallel with the cortical maturation of the individual, and therefore, like genes, they have an important role in the fate of the individual (normal aging process) [24, 25]. (4) Spindle density, frequency, or amplitude in EEG is closely related to cognition [22] and intelligence [23]. Considering the relationship of sleep spindles with critical concepts in human neurophysiology, such as understanding neuroplasticity, movement, memory and emotion control, cortical maturation, aging, cognition, and intelligence [26–28], the importance of SPINDILOMETER and the idea of developing new algorithms on it becomes more important. Recent studies have emphasized the importance of NREM 2 sleep and the deepening of sleep. Higher sleep spindle densities correlate with longer N2 sleep duration and greater resilience to external perturbation. Sleep spindles cluster on an infraslow time scale of ~ 50 s, thought to be correlated with periods of NREMS fragility. In some sleep-related movement disorders, involuntary limb movements occur periodically on this time scale [8, 36, 53]. Changes in the number and intensity of sleep spikes in the EEG may indicate a malfunction in the TRN-ThCx circuit. “Mood disorders, sleep movement disorders, cognitive deficits, attention and hyperactivity disorders, schizophrenia, epileptic seizures, Parkinson’s, Alzheimer’s, mental retardation, abnormal cortical maturation and recovery processes after brain stroke (neuroplasticity)” are the examples of TRN-ThCx circuit failures [8, 26–29]. SPINDILOMETER and its upgraded versions, which we are developing, may be pathognomonic in the diagnosis of these complex diseases in pediatric and adult patients and may become a good marker for following the treatment and progression of these patients.

### The relationship between EEG signal analysis and sleep spindle “a brief overview of recent literature”

German neurologist Hans Berger (1873–1941), considered as the father of human EEG recordings by distinguishing wake and sleep waves, Nobel Prize-winning British electrophysiologist Edgar Douglas Adrian (1889–1977) who noted that spindle rhythms could be recorded from the cut end of thalamocortical fibers in the cat, and Alfred Lee Loomis (1887–1975) were the turning points in sleep electrophysiology [53–55]. From the nineteenth century to the twenty-first century, this knowledge has translated into discoveries and technological advances in EEG for both the sleeping and the awake brain. Nowadays, the pace of sleep EEG studies has accelerated in an attempt to associate electrophysiologic activity to cognition and cognitive disorders. However, signal processing methods (appropriate calculation and interpretation of metrics) used in contemporary sleep EEG has gained importance. The ‘spindle,’ first described by electrophysiologist Edgar Douglas Adrian [53], has evolved thanks to computer and machine learning models. Sleep spindles can be detected automatically by computers, but the results need to be evaluated by visual-scoring experts and sleep physicians. Hours of scoring with the naked eye are laborious. Lacourse et al. analyzed sleep spindles using four important parameters—“the absolute sigma power, relative sigma power, sigma correlation, and sigma covariance”—on single-channel EEG [35]. Lacourse et al. achieved 68% sensitivity and 74% specificity in their study, while SPINDILOMETER achieved 92.4% sensitivity and 96.6% specificity. During our study on six different EEG channels, we applied four different feature extraction algorithms, namely (a) “pre-processing of EEG signals for SPINDILOMETER,” (b) “Power Spectral Density, Continuous Wavelet Transform, Non-Gaussianity Score, and Bispectrum Score Features (Xd),” which made us think that SPINDILOMETRE achieved higher diagnostic accuracy values. Adamczyk et al. used “Automatic Sleep Spindle Detection” in their study investigating sleep spindle differences in monozygotic and dizygotic twins. In the preprocessing stage of this model, the algorithm signal was resampled to 100 Hz to reduce the computational load [33]. Since SPINDILOMETER uses normalization in the preprocessing stage, the specificity and sensitivity values are thought to be higher than in this study. When we continue to examine recent machine-learning methods on EEG, Li et al. used a complex demodulation method to detect sleep spindle forms and calculate features after preprocessing [32]. Ahmed et al. detected sleep spindles with 93.7% accuracy by applying wavelet packet transform and Teager energy operator [56]. Mei et al. developed an automatic pipeline for sleep spindle classification using an optimized filter-based thresholding model [57]. Kinoshita et al. performed sleep spindle prediction using wavelet transform and random under-sampling boosting [58]. Kulkarni et al. proposed a deep-learning strategy (SpindleNet) to detect sleep spindles based on a single EEG channel [59]. The model SPINDILOMETER, which we created by analyzing six channels of EEG data in PSG, is more prominent than other models with its preprocessing, feature extraction, feature selection methods, and classification algorithms. SPIDILOMETRE is a fast scorer with high sensitivity (92.47%), specificity (96.60%), and accuracy (94.61%) using the latest signal processing methods (Table 6).

**Table 6:** The sensitivity (%), specificity (%) and accuracy (%) of the sleep spindle analysis performed in volunteers

	Sensitivity (%)	Specificity (%)	Accuracy (%)
Lacourse et al., 2019 [35]	68	74	–
Adamczyk et al., 2015 [33]	72	90	–
Li et al., 2017 [32]	–	–	76, 87, 90
Ahmed et al., 2009 [56]	–	–	93.7
Mei et al., 2017 [57]	–	–	61
Kinoshita et al., 2020 [58]	76.9	–	–
Kulkarni et al., 2019 [59]	97.7	–	–
Our study, 2024	92.47	96.60	94.61

### Reasons for developing the SPINDILOMETER

SPINDILOMETER was developed for two reasons: (1) for sleep and neurophysiology research and (2) for monitoring the diagnosis and treatment of neuropsychiatric disorders.

As demonstrated in this paper, sleep spindles provide us with very important clues about the physiological mechanisms in the TRN-ThCx neuronal circuits in the brain and the disorders that occur when these circuits malfunction. SPINDILOMETER is an algorithm compatible with this important EEG waveform. For us, the reason for creating and further developing a SPINDILOMETER is that (a) physicians dealing with neurological sciences tend towards accurate diagnoses, and (b) brain researchers like using an algorithm that has reached high diagnostic accuracy values in their articles.

### Why PSG^+^ SPINDILOMETER?

PSG systems in the hospital sleep laboratories provide physicians and researchers important information regarding sleep physiology and diseases. Sleep spindles, as the building blocks of the microstructural architecture of EEG, are routinely monitored for PSG systems. However, when we look at the content of PSG reports created for physicians, no data are available on sleep spindles. We would like to see our algorithm included in PSG systems, and we believe that PSG^+^ SPINDILOMETER is necessary for both patients and physicians and scientific research. In our opinion, while PSG applications and the resulting reports, through the criteria determined by the AASM, provide us information about the brain and bodily functions after a night’s sleep (at least 6 h), they leave the interpretation of very important information incomplete. This information is, of course, related to the physiology and diseases of the TRN-ThCx region. This lack of information in PSG reports can be eliminated by means of SPINDILOMETER by AASM criteria. Moreover, this information can be improved over time, and its scope can be expanded because, based on their number and density SPINDILOMETER algorithms are expected to predict the physiological mechanisms that are disrupted while detecting sleep spindles and making comments about sleep physiology and disorders, as well as cognitive, emotional, and movement disorders.

### Limitations of the study and future work for improving SPINDILOMETER

Our main goal in this study was not solely to develop an algorithm that successfully calculates the number and density of sleep spindles. We also took into account the microstructures (sleep spindles, K-complexes) that form the architecture of sleep EEG and the arousals that occur during sleep; we wanted to create sleep maps of individuals that allow us to interpret their interactions with one another. From this point of view, the current study is incomplete; we can even in our opinion that we are in the preliminary stages of our studies to reach the sleep maps we want. The strength of our study is having more subjects compared to the literature. However, our limitation is that these subjects were individuals diagnosed with sleep-disordered breathing. Computer analysis of the micro-elementary electrophysiological signals of sleep EEG in undiagnosed children, young individuals, and healthy adults will provide us with more information about normal functioning. Finally, we know and wish that the idea of combining signal processing methods with new neuroimaging techniques to monitor the effects of sleep spindles on TRN-ThCx and cortex will lead us to algorithmic versions of “SPINDILOMETER^+^ Neuroimaging” in the future.

## Conclusion

SPINDILOMETER can be incorporated into PSG analysis conducted in sleep laboratories. Thus, this model provides diagnostic convenience to the physician in understanding the neurological events associated with sleep spindles and sheds light on thalamocortical region research in the fields of neurophysiology and electrophysiology.

## Data Availability

The datasets analyzed during the current study are not publicly available due to the policy of the project agreement but are available from the corresponding author upon reasonable request.
